# Congenital Mpox Syndrome (Clade I) in Stillborn Fetus after Placental Infection and Intrauterine Transmission, Democratic Republic of the Congo, 2008

**DOI:** 10.3201/eid2911.230606

**Published:** 2023-11

**Authors:** David A. Schwartz, Placide Mbala-Kingebeni, Kerry Patterson, John W. Huggins, Phillip R. Pittman

**Affiliations:** Perinatal Pathology Consulting, Atlanta, Georgia, USA (D.A. Schwartz);; Institut National de Recherche Biomédicale, Ministère de la Santé Publique, Kinshasa, Democratic Republic of the Congo (P. Mbala-Kingebeni);; US Army Medical Research Institute of Infectious Diseases, Fort Detrick, Maryland, USA (K. Patterson, J.W. Hudgins, P.R. Pittman)

**Keywords:** mpox, congenital mpox, monkeypox virus, clade I, viruses, stillbirth, pregnancy, autopsy, poxvirus, pathology, congenital infection, placenta, Democratic Republic of the Congo, Africa

## Abstract

We report the autopsy pathology findings of a 21-week stillborn fetus with congenital mpox syndrome that occurred in the Democratic Republic of the Congo in 2008. The fetus acquired mpox from the mother after intrauterine transplacental monkeypox virus transmission. We confirmed monkeypox virus infection in the mother, fetus, and placenta by using a monkeypox virus–specific quantitative PCR. Subtyping of the virus was not performed, but the mother and fetus were almost certainly infected with the clade I variant that was endemic in the Democratic Republic of the Congo at the time. Risk for intrauterine infection appears to differ between virus clades, but clinicians should be aware of potential for intrauterine monkeypox virus transmission among pregnant persons during ongoing and future mpox outbreaks.

Monkeypox virus, which causes mpox, is the most medically concerning member of the genus *Orthopoxvirus*. Monkeypox virus is related to the variola virus, the etiologic agent of smallpox before its eradication. Mpox has caused illness and death in endemic countries of Central and West Africa, where it infects thousands of persons annually. In 2022, a global outbreak of mpox occurred that has resulted in >87,000 infections, most of which have occurred in nonendemic countries (*1*). We describe a case of stillbirth caused by congenital mpox after transplacental transmission that occurred in the Democratic Republic of the Congo (DRC) in 2008. Although some aspects of this case have been previously reported (*2*–*4*), the onset of the 2022–2023 global mpox outbreak prompted reevaluation of the case for additional placental and perinatal pathology information.

## Methods

To examine the clinical and epidemiologic aspects of monkeypox virus, the Institut National de Recherche Biomédicale (Kinshasa, DRC) and the US Army Medical Research Institute of Infectious Diseases jointly designed and conducted a prospective study at the Kole Hospital in Kole, located in the Sankuru District of Kasaï-Oriental Province in DRC (*2*). The Kole Hospital is the sole health facility in the region where persons infected with mpox are hospitalized and treated. The region is rural and consists of areas of savanna and tropical rainforest with interspersed traditional agricultural fields. The inhabitants are members of a subtribe of the Nkutu (or Okutshu) ethnic group and are mostly hunters and subsistence farmers who depend on wildlife, predominantly monkeys and rodents, for their main source of protein. The inhabitants reside in small villages of <100 persons in clearings and have a communal lifestyle composed of extended family groups of <15 persons living in simple wattle and daub houses. Because participants for the study were not actively recruited, some mpox cases might have occurred in the region but not been included in the study, and other cases might not have been detected because the relative isolation of the region limits access to medical care and hospitalization.

As part of the original study, in 2008, a 22-year-old G3P2 pregnant person was seen at 19 weeks’ gestation. She was well-nourished and had a positive malaria status but had no allergies or obstetric-related underlying conditions. At enrollment, the mother had moderate mpox with a maculopapular rash and lesion count of 113, accompanied by fever and submandibular lymphadenopathy, but no genital lesions. Mpox infection was confirmed by quantitative PCR (qPCR). She had become febrile at 18 weeks’ gestation and had mpox viremia confirmed by virus-specific qPCR. Two weeks after enrollment, she noted absence of fetal movement, which was confirmed both clinically and by 2 sequential obstetrical ultrasounds, leading to diagnosis of intrauterine fetal death. 

Maternal viremia rose rapidly from 10^2^ to 10^6^ viral copies/mL over a 3-day period after cessation of fetal movement. Transcutaneous amniocentesis was positive for mpox virus. Labor was induced by using oxytocin. After membrane rupture, a 21-week gestation stillborn female fetus was delivered vaginally. Umbilical vein blood was positive for mpox virus by qPCR. Oral and written autopsy consent was obtained in the native language from the mother, per the study protocol, which included photography and use of all body specimens, including tissue samples, for further study and publication. After the delivery, the mother was monitored in the hospital; although depressed, she recovered from the mpox infection and was discharged to home in good health. She was well when she returned for her 75-day postpartum follow-up visit.

### Fetal Autopsy

The fetal autopsy was performed in a surgical operating suite at the Hôpital General du Référence in Kinshasa. The lighting was suboptimal for photography, and equipment for measuring organ weights and taking dictation was not available. Photographs of the fetus were taken by using a digital Stylus 790 SW camera (Olympus, https://explore.omsystem.com).

**Figure 1:**
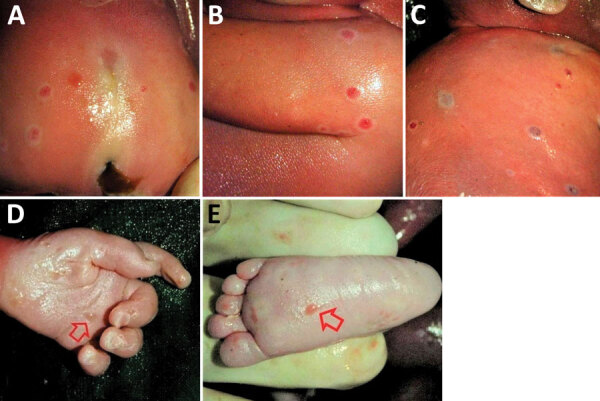
Cutaneous lesions on a stillborn fetus with congenital mpox after placental monkeypox infection and intrauterine transmission, Democratic Republic of the Congo, 2008. A) Buttocks; B) right upper arm; C) right shoulder and back; D) palm of left hand (arrow); E) plantar surface of left foot (arrow).

**Figure 2:**
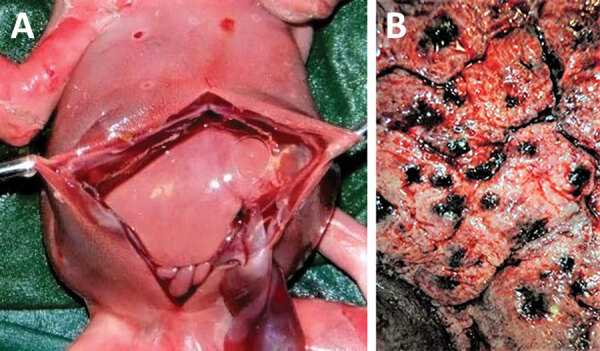
Evidence of congenital mpox syndrome from a stillborn fetus after placental monkeypox infection and intrauterine transmission, Democratic Republic of the Congo, 2008. A) Marked hepatomegaly demonstrating liver twice normal size for gestational age, ascites, and hydrops. B) The maternal surface of the placenta had numerous discrete punctate hemorrhages.

The female fetus was macerated and had an estimated weight of 300–350 g. Body measurements were crown-heel length 27 cm, crown-rump length 16 cm, arm span 20 cm, head circumference 17–18 cm, and abdominal circumference 30 cm. The outer canthal distance and interpupillary distance were normal. No evidence of either symmetric or asymmetric fetal growth restriction was noted. The fetus displayed nonimmune hydrops fetalis, an abnormal accumulation of fluid in >2 body areas. The eyelids were closed and appeared swollen and edematous. The skin of the scalp and face showed multiple distinctive pale pink to white maculopapular lesions (pox) involving the right nostril, upper mid-lip, forehead, right cheek, and temporoparietal regions of the scalp. The lesions on the head were not as well developed as lesions on other parts of the body. Many maculopapular lesions, varying from 0.3–0.5 cm in size, were noted on the skin of the extremities, including the soles of the feet and palms of the hands bilaterally, back, chest, shoulders, abdomen, and buttocks (Figure 1). Many of the lesions were bright red surrounded by white halos, and some had superficial ulcerations. Results of internal organ examination were unremarkable except for the liver, which demonstrated hepatomegaly and measured 8 × 6 × 4 cm (Figure 2, panel A). No pox lesions were noted on any surfaces of the internal organs of the chest or abdomen. Ascites was present and was sampled for viral analysis. The placenta was examined at the time of autopsy, was of normal shape, and weighed ≈250 g. The maternal surface was abnormal, showing multiple punctate hemorrhages varying from 0.4–0.8 cm in diameter (Figure 2, panel B). The amniotic surfaces of the umbilical cord, extraplacental membranes, and placental disc had no pox-like lesions.

### Microscopic Findings

**Figure 3:**
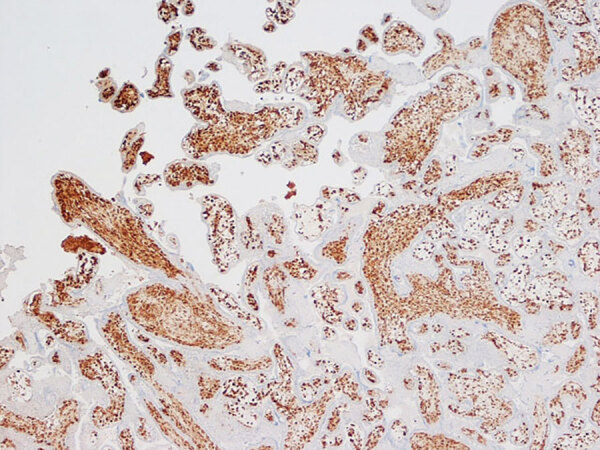
Immunohistochemistry of placenta from a stillborn fetus after placental monkeypox infection and intrauterine transmission, Democratic Republic of the Congo, 2008. Microscopic findings show diffuse and intense positive staining for orthopoxvirus antigen in Hofbauer cells in the chorionic villi. Immunohistochemistry with antibody to vaccinIa virus counterstained with hematoxylin and eosin. Original magnification ×10.

Because fetal organs had extensive postmortem autolysis, the only slides available for histological examination were from the fetal skin, liver, and placenta. We stained those formalin-fixed specimens by using immunohistochemistry with vaccinia virus antibodies. Because vaccinia virus is also an orthopoxvirus agent, its antibodies have strong cross-reactivity to monkeypox virus. Strong immunohistochemical positivity for poxvirus was detected in the skin, liver, and placental tissues. In particular, the placenta was reevaluated by a perinatal pathologist during the global 2022–2023 mpox outbreak. It demonstrated extensive and diffuse positive staining of villous stromal cells that were consistent with Hofbauer cells, the native population of villous macrophages (Figure 3). Those cells were increased in number within the chorionic villi, a finding termed Hofbauer cell hyperplasia. We searched for Guarnieri bodies, intracytoplasmic inclusions consisting of aggregates of orthopoxvirus virions often seen in infected epithelial cells, but none were definitively identified.

### Viral Analysis

Results of quantitative PCR testing for monkeypox virus were positive from multiple samples: fetal organs, skin lesions, sterile peritoneal fluid, umbilical cord blood, and placenta. Tissue obtained from the fetus at autopsy had 1.7 × 10^7^ genome copies/mL, the placenta had 2.4 × 10^7^ genome copies/mL, and fetal blood obtained from the umbilical cord vein blood had 2.5 × 10^7^ genome copies/mL. During autopsy, sterile peritoneal fluid was obtained from the fetus and found to contain 1.6 × 10^3^ genome copies/mL of monkeypox virus. Although subtyping of the virus was not performed, the mother and fetus were almost certainly infected with the clade I mpox variant that was endemic in DRC at the time.

## Discussion

Most of our knowledge of the effects of mpox in pregnancy is derived from investigations reported as part of the Kole Human Monkeypox Infection Study in the Sankuru Province of DRC during March 2007–July 2011 (*2*,*3*). Among a cohort of 222 symptomatic mpox patients (36% female, 64% male) who were seen at the General Hospital of Kole in DRC, 4 were pregnant persons with mpox infection. One pregnancy resulted in birth of an uninfected healthy infant, 2 pregnancies resulted in first trimester miscarriage, and the fourth pregnancy (described in this report) resulted in intrauterine fetal death at 21 weeks’ gestation. Unfortunately, no information is available on the other 2 stillborn fetuses, except that both mothers had active mpox disease. 

A case of suspected congenital mpox occurred in DRC in the 1980s, in which a pregnant woman developed a rash, was confirmed to have mpox, and subsequently delivered a live-born 24-week gestation neonate, who at the time of delivery exhibited a generalized rash that was consistent with mpox (*5*). In Nigeria, fetal losses associated with maternal mpox infection during 2017–2018 were reported at 16- and 21-weeks’ gestation, but no fetal studies were performed (*6*,*7*).

The microscopic findings in the placenta of our case were remarkable, consisting of diffuse and intense immunohistochemical positivity for orthopoxvirus antigen in villous stromal cells that were consistent with Hofbauer cells and virus-positive staining in the skin and liver. No definitive Guarnieri bodies were identified. Unlike in cases of smallpox, in which those intracytoplasmic aggregates of viral material often were identified, much less is known regarding their occurrence in monkeypox-infected tissues, but similar structures have been identified in infected cutaneous lesions (*8*).

This report adds additional details to the previously published autopsy pathology findings on a stillborn fetus with confirmed congenital mpox syndrome (*2*,*4*). This case report adds new information on confirmed fetal intrauterine mpox infection and serves as an example of the characteristic macroscopic presentation of congenital mpox. The occurrence of cutaneous pox lesions diffusely extant on all parts of the body is similar that those seen in cases of congenital smallpox infection before its eradication (*4*). The finding of poxvirus antigen staining within the chorionic villi of the placenta in this stillborn fetus is indicative of intrauterine transplacental transmission and is similar to that seen in placentas from viral TORCH (an acronym for toxoplasmosis, other agents, rubella, cytomegalovirus, and herpes simplex) infections, including cytomegalovirus, Zika virus, and SARS-CoV-2 (*9*–*11*). As with those viral diseases, the chorionic villi of the placenta in our case were found to be infected, providing a potential pathway for crossing the maternal–fetal interface. The death of the fetus in this case was the result of infection with monkeypox virus clade I (formerly the Congo Basin clade). Risk for adverse perinatal outcomes appears to differ depending on virus clades. According to published literature, clade I virus has a 75% perinatal fatality rate (*2*). In contrast, at least 58 cases (likely more) of pregnant women infected with mpox occurred during the 2022–2023 global mpox outbreak, but no confirmed cases of fetal infection or intrauterine transmission were reported (*4*,*12*,*13*). 

Phylogenetic analysis identified the 2022–2023 mpox outbreak strain to be an offshoot of the clade II (West African) virus and that it had sufficient novel mutations to be classified as a new subclade, clade IIb (*14*). The absence of perinatal disease from clade IIb corresponds to the <0.1% overall case-fatality rate among nonpregnant persons (*13*,*15*). Clade IIb has produced less severe disease than have the mpox clade I or IIa variants (*13*), but the large predominance of men who have sex with men that were infected during the 2022–2023 outbreak might also play a role. The difference in perinatal death between the various mpox clades might be analogous to the differences in the frequency of miscarriage and stillbirth associated with differing variants of SARS-CoV-2 during the COVID-19 pandemic (*16*).

In conclusion, we report a case of fetal death after placental infection and intrauterine transmission of monkeypox virus clade I in the Democratic Republic of the Congo in 2008. Risk for intrauterine infection appears to differ between virus clades. Nonetheless, clinicians should be aware of potential for placental infection and intrauterine transmission of monkeypox virus among pregnant persons during ongoing and future mpox outbreaks.
